# Design of a novel 5‐camera surface guidance system with multiple imaging isocenters

**DOI:** 10.1002/acm2.13750

**Published:** 2022-08-10

**Authors:** Ozgur Ates, Li Zhao, David Sobczak, Fakhriddin Pirlepesov, Chia‐ho Hua, Ben Waghorn, Thomas E. Merchant

**Affiliations:** ^1^ Department of Radiation Oncology St. Jude Children's Research Hospital Memphis Tennessee USA; ^2^ Vision RT Inc. London UK

**Keywords:** multiple imaging isocenters, proton therapy, SGRT, surface‐guided radiation therapy

## Abstract

**Purpose/objective(s):**

Surface‐guided radiation therapy (SGRT) can track the patient surface noninvasively to complement radiographic image‐guided radiation therapy with a standard 3‐camera system and a single radiation/image isocenter. Here we report the commissioning of a novel SGRT system that monitors three imaging isocenters locations in a proton half‐gantry room with a unique 5‐camera configuration.

**Materials/methods:**

The proton half‐gantry room has three image isocenters, designated ISO‐0, ISO‐1, and ISO‐2, to cover various anatomical sites via a robotic ceiling‐mounted cone‐beam CT. Although ISO‐0 and ISO‐1 are used to image the cranium, head and neck, and thoracic regions, ISO‐2 is often used to image body and extremity sites and contiguous craniospinal target volumes. The five‐camera system was calibrated to the radiographic isocenter by using a stereotactic radiosurgery cube phantom for each image isocenter.

**Results:**

The performance of this 5‐camera system was evaluated for 6 degrees of freedom in three categories: (1) absolute setup accuracy relative to the radiographic kV image isocenter based on the DICOM reference; (2) relative shift accuracy based on a reference surface capture; and (3) isocenter tracking accuracy from one isocenter to another based on a reference surface capture. The evaluation revealed maximum deviations of 0.8, 0.2, and 0.6 mm in translation and 0.2°, 0.1°, and 0.1° in rotation for the first, second, and third categories, respectively. Comparing the dosimetry and latency with static and gated irradiation revealed a 0.1% dose difference and positional differences of 0.8 mm in *X* and 0.9 mm in *Y* with less than 50 ms temporal accuracy.

**Conclusion:**

The unique 5‐camera system configuration provides SGRT at the treatment isocenter (ISO‐0) and also imaging isocenter locations (ISO‐0, ISO‐1, and ISO‐2) to ensure correct patient positioning before and after radiographic imaging, especially during transitions from the offset imaging isocenters to the treatment isocenter.

## 1 INTRODUCTION

Surface‐guided radiation therapy (SGRT) uses emerging technologies to facilitate non‐radiographic localization in radiation oncology. With the commercial products now available, SGRT is playing an important role in enhancing the quality of radiation treatments and patient safety as a complement to conventional image‐guided radiotherapy (IGRT) systems. Standard IGRT procedures, using 2D orthogonal imaging with kilovoltage (kV) or megavoltage (MV) X‐rays, enable the daily alignment of images to the patient reference computed tomography (CT) via bony anatomy, fiducial marks, and soft tissue. The advent of cone‐beam CT (CBCT)^1^, ^2^, ^3^ has improved the setup accuracy of the image guidance process through using volumetric CBCT imaging for daily localization to the treatment isocenter.

SGRT offers particular benefits for pediatric patients. Both awake and sedated children can benefit from noninvasive localization and monitoring technology. Awake children may be more susceptible than adults to noise and the busy environment; therefore, they may not be compliant and may move during imaging and delivery. SGRT can potentially reduce imaging time during setup and can also help monitor the patient surface throughout treatment delivery. Younger children are generally sedated for radiation therapy for various reasons, mostly related to immaturity, the environment, immobilization devices, and pain.^4^ Prolonged anesthesia because of repeated imaging may introduce additional risks, including that of secondary malignancies. SGRT may facilitate a reduction in the number of imaging sessions and, hence, reduce the imaging dose and anesthesia time by providing surface alignment before radiographic imaging. Children's Hospital Los Angeles reported a workflow in which skin marks and tattoos were eliminated with SGRT.^5^, ^6^

Proton therapy introduces special challenges because the proton Bragg peak is sensitive to changes in water equivalent thickness (WET) in the beam path.^7^ Protons are fundamentally different from photons, and their use requires more attention to tissue changes. In intensity‐modulated proton therapy, robust treatment planning considers setup and proton range uncertainties, to some extent, to accommodate interfractional setup variations and WET changes from the source to the target volumes. However, there may still be interfractional and intrafractional errors, including deformations, tissue changes, and respiratory motion, that result in a significant interplay effect between the target motion and beam delivery for protons.^8^ Methods have been proposed to mitigate such interplay effects during lung treatments by using gating and rescanning.^9^

In some proton centers, a standard 3‐camera SGRT system may not work because of the complexity of the vault design. Gantry motion or imaging equipment in the proton vault may block the view of the SGRT camera positions, causing suboptimal operation of the SGRT system. One proton center had to relocate the SGRT cameras to a nonsymmetric arrangement,^10^ whereas another group reported that the camera placement was dictated by the design of the proton vault.^11^ The Maastro Clinic proton therapy center (Maastricht, the Netherlands) had to install a nonstandard SGRT camera system because the image isocenter was located away from the treatment isocenter for CBCT imaging. In this case, a fourth camera was needed to focus on the CBCT imaging position.^12^

Here we provide the commissioning report for a novel 5‐camera SGRT system that was designed and installed in our proton half‐gantry room; however, the design concept can be translated into any photon or proton clinic needing to improve their SGRT systems because of multiple image isocenters or obstruction of the fields of view of the SGRT cameras. To our knowledge, this report is the first to present the performance of a nonstandard SGRT system with more than three cameras that have been used in pediatric proton therapy.

## 1 METHODS

This section describes the design and commissioning of this unique 5‐camera SGRT system in a proton half‐gantry room with three image isocenters, including the in‐room survey, simulation study, and installation (Section 2.1); the integration of SRS (stereotactic radiosurgery) radiographic image calibration (Section 2.2); the end‐to‐end commissioning workflow (Section 2.3); the routine physics quality assurance (QA) implementation (Section 2.4); and the dynamic gating tests (Section 2.5).

### 1.1 The in‐room survey, simulation study, and installation

An engineering survey was conducted by Vision RT (Vision RT Inc., London, UK) in a Hitachi half‐gantry room (PROBEAT‐V, Hitachi Ltd., Tokyo, Japan). The necessary physical measurements were simulated in a vendor‐based proprietary software to find the best locations for the camera placements. Initially, the standard 3‐camera configuration was simulated; however, because of the complexity of the three distinct image isocenters, a 5‐camera pod system was found to be the ideal solution. Table 1 shows the physical distances of the camera positions with respect to the three image isocenters in room coordinates.

**TABLE 1:** Survey measurements for the 5‐camera system relative to the three image isocenters

Pod	Pod center in *X* (mm)	Pod center in *Y* (mm)	Pod center in *Z* (mm)	Distance to Iso‐0 (mm)	Distance to Iso‐1 (mm)	Distance to Iso‐2 (mm)
1	435	−2114	−1319	2529	2590	3217
2	−2031	−1678	−1463	3013	2838	3403
3	−1588	758	−1420	2261	2080	2144
4	445	−1917	−1393	2411	2475	3263
5	−1789	345	−1424	2312	2110	2378

**FIGURE 1:**
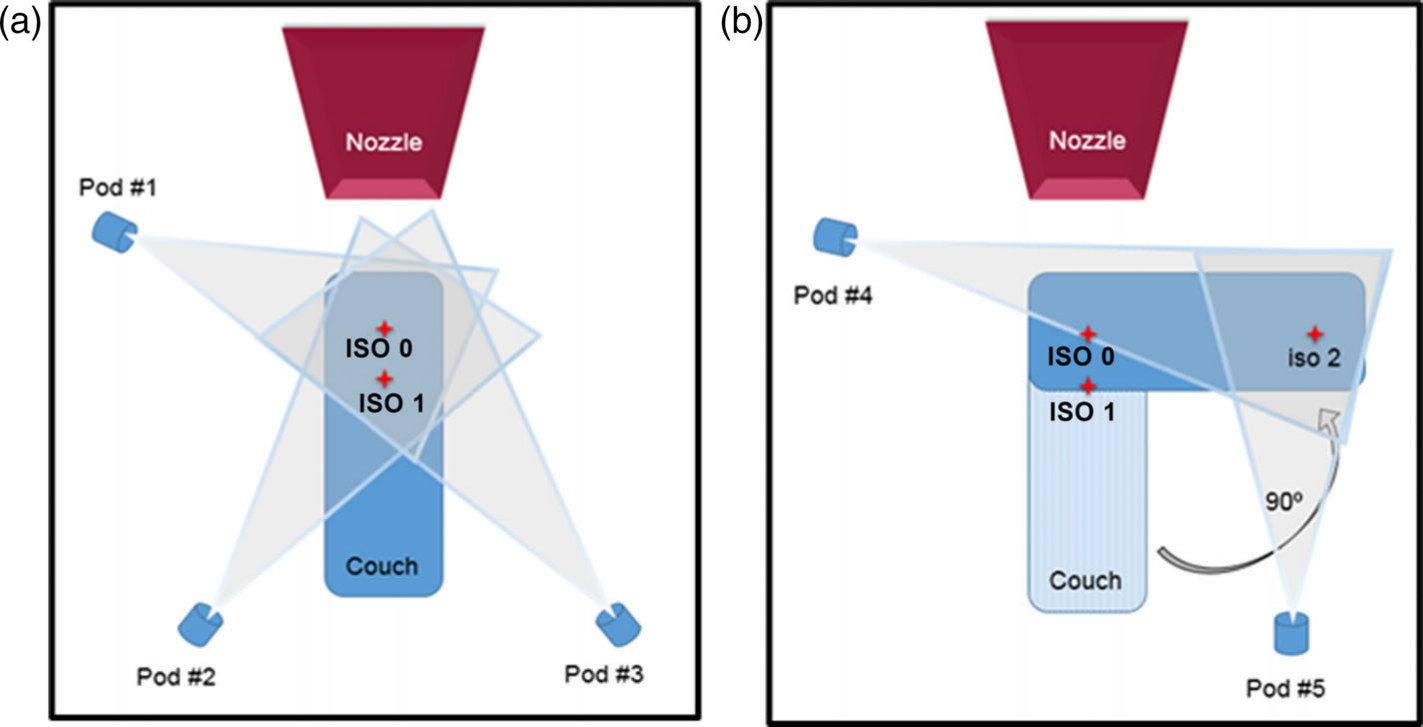
Camera placements or pods for the 5‐camera system that contains a 3‐camera system (a) covering ISO‐0 and ISO‐1 and a 2‐camera system, (b) covering ISO‐2 in the single proton room with three image isocenters

**FIGURE 2:**
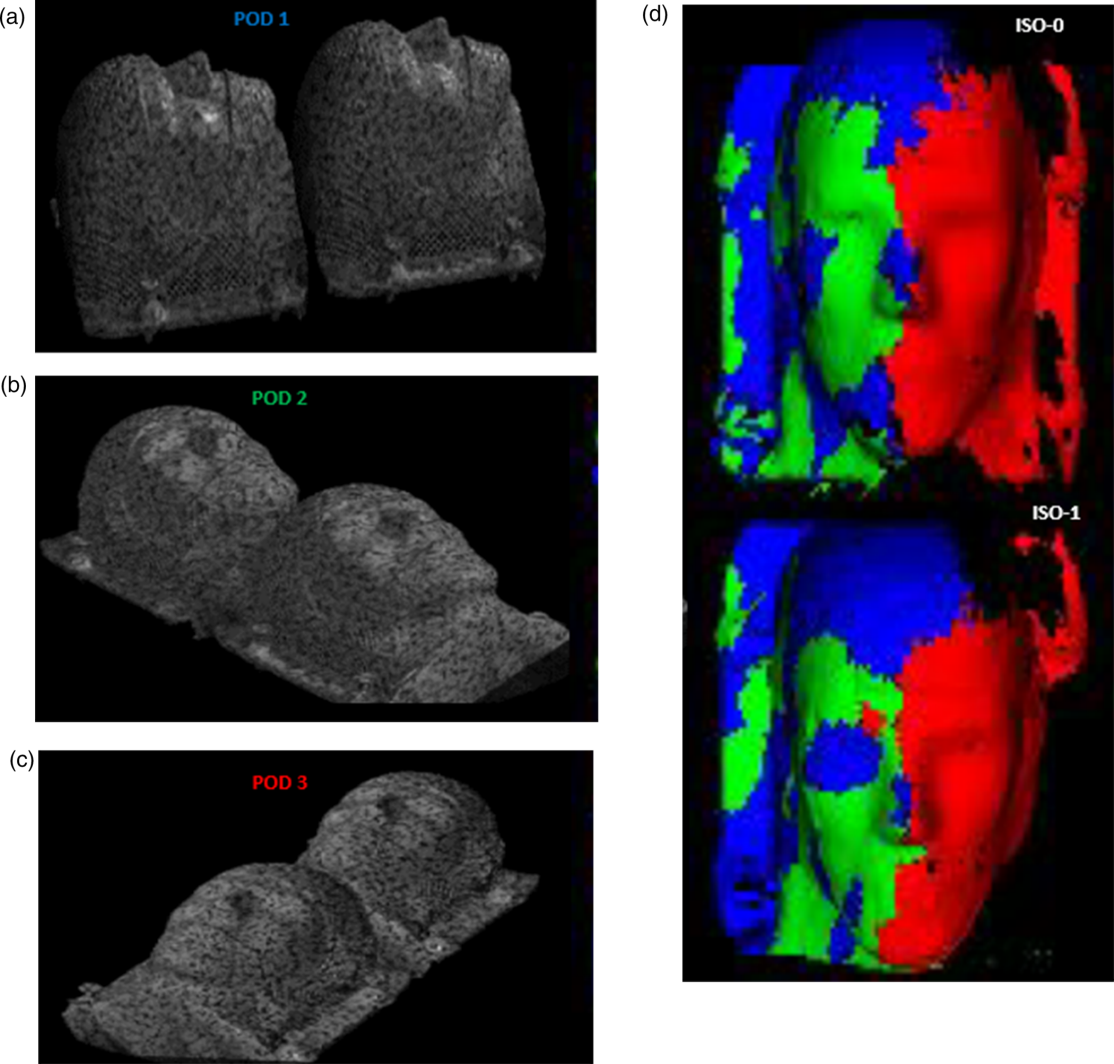
(a)–(c) Camera pod projections on the phantom at ISO‐0 and ISO‐1 with (a) pod 1, (b) pod 2, and (c) pod 3. (d) Composite color‐coded surface image merging the results for pod 1 (blue), pod 2 (green), and pod 3 (red)

**FIGURE 3:**
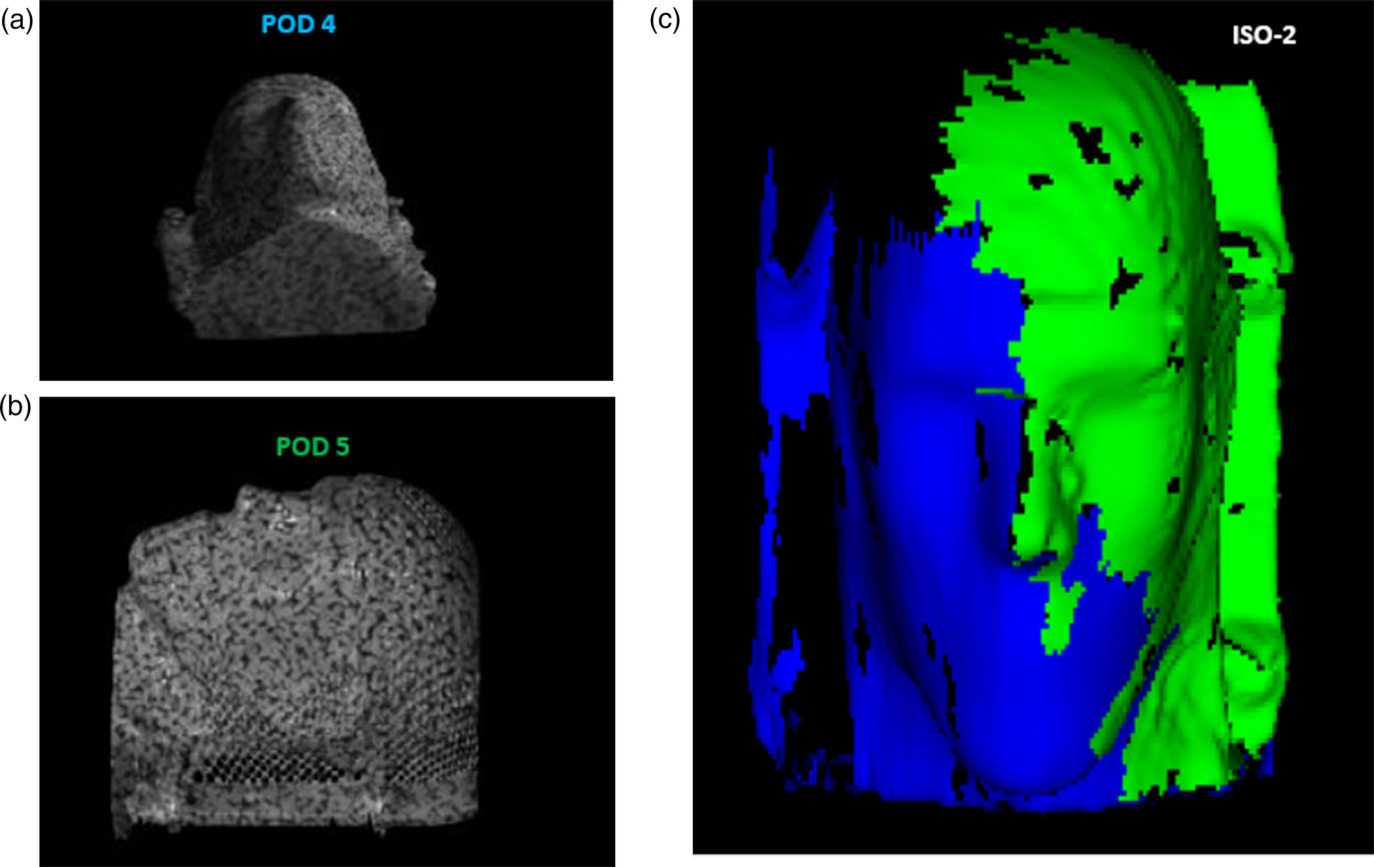
(a and b) Camera pod projections on the phantom at ISO‐2 with (a) pod 4 and (b) pod 5. (c) Composite color‐coded surface image merging the results with pod 4 (blue) and pod 5 (green)

The three image isocenters, namely, ISO‐0, ISO‐1, and ISO‐2, are the kV‐CBCT image isocenters for the ceiling‐mounted robotic C‐arm (Hitachi Ltd., Tokyo, Japan), whereas ISO‐0 is the only radiation isocenter. ISO‐1 is 27 cm from ISO‐0 on the longitudinal axis, and IS0‐2 is 100 cm from ISO‐0 on the lateral axis. Figure 1 shows an overhead schematic view of the camera placements in a non‐scale representation. In this unique configuration, 3‐camera pods (#1, #2, and #3) cover the image isocenters of ISO‐0 and ISO‐1 and the other 2‐camera pods (#4 and #5) cover the image isocenter of ISO‐2. The simulation study was completed using a vendor‐based proprietary software to inspect visually the surface coverage on the phantom. The study served two purposes: (1) to view each camera projection on the phantom by using the speckle pattern, and (2) to determine the contribution of each camera pod to the phantom surface coverage by using the 3D color‐coded surface function. The simulation was conducted for all three image isocenters with the 5‐camera system, that is, with a 3‐camera system serving IS0‐0 and ISO‐1 and a 2‐camera system covering only ISO‐2. The dark regions that were not seen by any camera pod were determined to be the “blind spots” of the 5‐camera system. Figure 2 shows the speckle patterns from each camera projection from the 3‐camera system (pods 1–3) on the phantom for ISO‐0 and ISO‐1, which are 27 cm apart on the longitudinal axis. It also presents the composite surface coverage when the results are merged to form a single‐phantom surface image at isocenters 0 and 1. Figure 3 demonstrates the similar speckle patterns obtained with each camera projection from the 2‐camera system on the phantom for ISO‐2, which is 100 cm from ISO‐0 on the horizontal axis. Also shown is the composite surface coverage obtained when the projections from pods 4 and 5 are integrated at ISO‐2.

**FIGURE 4:**
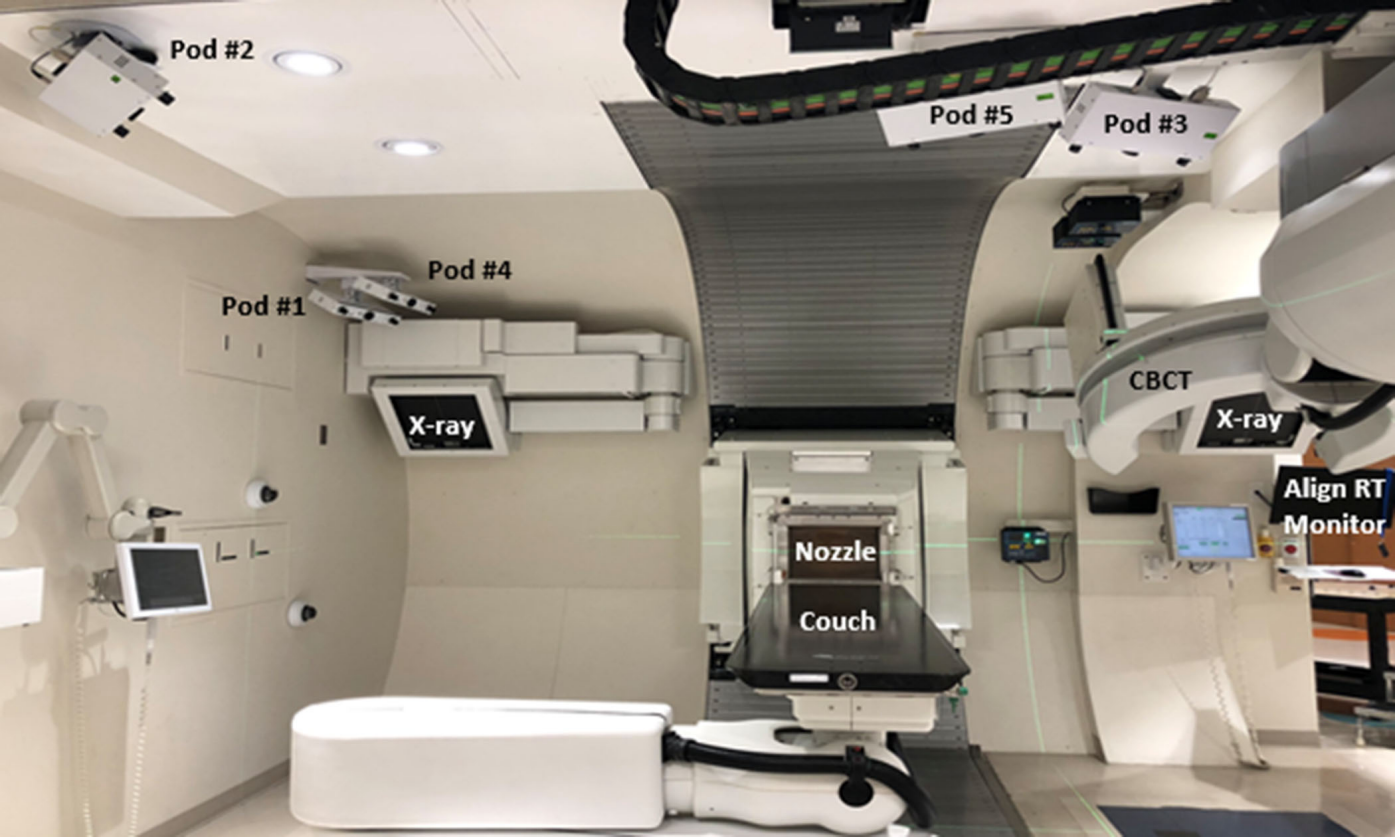
The proton half‐gantry room with its busy ceiling space after the installation of the 5‐camera system

During the installation, the large physical distances between the image isocenters and the pathways of the robotic kV‐CBCT robotic C‐arm, the gantry rotation, and the wall‐mounted orthogonal X‐ray panels all contributed to the complexity of the camera placements and resulted in limitations of the fields of view of the camera pods (noted as dark regions in the simulation study). Figure 4 shows the in‐room placements of the 5‐camera system in the already occupied ceiling of the proton half‐gantry room with the couch at 270°, the gantry at 90°, and the C‐arm in the parked position.

### 1.2 Integrating the SRS radiographic image calibration

After being installed in the proton room, the 5‐camera system was separated into a 3‐camera system and a 2‐camera system that were connected to two separate computer boxes but shared the same patient database. The calibration isocenters of the 3‐ and 2‐camera systems were designated ISO‐0 and ISO‐2, respectively. For the two systems, the standard AlignRT plate calibration was performed at ISO‐0 and ISO‐2 separately. For ISO‐1, the “move isocenter” function was used simply to shift the image isocenter by 27‐cm longitudinally. Volumetric CBCT imaging is a routine practice for daily patient positioning in our clinic; thus, it is important for us to verify congruence between the radiographic imaging isocenter and the surface guidance isocenter. Vision RT offers a solution for this type of calibration called “SRS module,” which provides submillimetric calibration accuracy based on the radiographic isocenter of the CBCT imaging system. The module came with an SRS cube phantom with five radio‐opaque spheres with known coordinates distributed around the cube and dedicated software to analyze the 2D images acquired by the C‐arm CBCT and find the relevant shifts needed to move the new isocenter to the calibrated radiographic isocenter of the system.

Unfortunately, the standard SRS module was incompatible with the Hitachi C‐arm CBCT because of collimation issues on the software side. An in‐house software was developed to eliminate the inconsistencies between the two systems during post‐processing. Whenever the 2D image was acquired with the C‐arm CBCT, the in‐house software was initiated to truncate the images to exactly 5 cm × 5 cm for the use in calculating the congruence between the laser‐based standard plate calibration and the radiographic image calibration.

For radiographic calibration, the images from all four cardinal angles, namely, 0°, 90°, 180°, and 270°, were needed at both the ISO‐0 and ISO‐2 imaging isocenters. Once these four 2D images had been acquired, the calibration algorithm in the SRS module could calculate the shifts necessary to make the isocenter in AlignRT coincide with the isocenter of the imaging system.

### 1.3 The end‐to‐end commissioning workflow

Because of the complexity of the three image isocenters, each isocenter was expected to satisfy the clinical needs of the SGRT program in the half‐gantry proton room. After the 5‐camera system was calibrated to the radiographic image isocenters, a series of commissioning objectives were pursued for each isocenter using the SRS cube phantom as indicated in the following:

1. Absolute setup accuracy: The real‐time delta (RTD) values on the AlignRT monitor as the difference between the DICOM reference and the current surface image in 6 DoF, after CBCT‐guided image registration was completed.
2. Relative shift accuracy: The RTD values on the AlignRT monitor after couch was moved by known amounts in 6 DoF, based on the reference surface capture in AlignRT.
3. Tracking accuracy from one isocenter to another: The surface of the SRS cube was tracked among the image isocenters, for example, from ISO‐2 to ISO‐0 and from ISO‐1 to ISO‐0, based on the reference surface capture at the initial location. The final RTD values in 6 DoF was recorded at the destination isocenter.

For the reference CT scan, an IQon spectral CT imager (Philips Healthcare, Best, the Netherlands) was used to image the phantom. The surface contour of the DICOM image of the phantom was drawn in an Eclipse v13.7 treatment planning system (TPS) (Varian, Palo Alto, CA). The end‐to‐end commissioning process was as outlined in the following steps, which were followed for each isocenter, using the SRS cube phantom:

1. Scan the SRS cube phantom in the CT scanner and draw the surface contour in the TPS.
2. Export the DICOM image and structure into AlignRT.
3. Place the SRS cube at the imaging isocenter in the room, acquire CBCT images, and perform image registration.
4. Apply imaging shifts based on the DICOM reference image and record the RTD values (absolute setup accuracy).
5. Move the couch by known amounts and record the shifts in the RTD monitor (*relative shift accuracy*).
6. Track the SRS cube from one isocenter to another and record the RTD values (*tracking accuracy*).

### 1.4 Routine physics quality assurance implementation

The routine physics QA was performed according to the recommendations in the report of AAPM Task Group 147 (TG‐147) titled “Quality assurance for non‐radiographic radiotherapy localization and positioning systems.” Based on that report, the routine daily, monthly, and annual QA procedures were developed and tested for this SGRT system in the half‐gantry room.

For daily QA, apart from the vendor‐based daily plate calibration, a home‐made daily QA phantom that has been used for regular proton daily QA purposes was used to track the daily known shifts based on the reference capture of the phantom at ISO‐0 and ISO‐2, which are the calibration isocenters for the 5‐camera system. In this daily SGRT localization workflow, the surface capture of the shifted phantom is compared to the reference capture of the nominal phantom position to reveal the known translational shifts (tolerance = 2 mm) between two positions of the phantom immediately before daily CBCT imaging QA. The C‐arm of the CBCT does not block the cameras, whereas it is in the lateral positions at 90° or 270°. For the monthly QA, in addition to the vendor‐based monthly plate calibration, the SRS cube phantom was also used to reveal the absolute shift accuracy, using full‐rotation CBCT and image registration based on the reference DICOM image with ISO‐0 and ISO‐2 as the calibration isocenters. After the shifts from the image registration were applied, the RTD values were kept below 2 mm in translation and 1° in rotation.

For the annual QA, a full‐blown SRS module calibration was performed for each isocenter, that is, ISO‐0, ISO‐1, and ISO‐2. Thermal drift and reproducibility tests were also performed for each camera system. Dynamic gating tests were completed to achieve dosimetric accuracy within 2% and temporal accuracy (latency) within 100 ms at the radiation isocenter of ISO‐0, as discussed in the next section.

### 1.5 Dynamic gating tests

**FIGURE 5:**
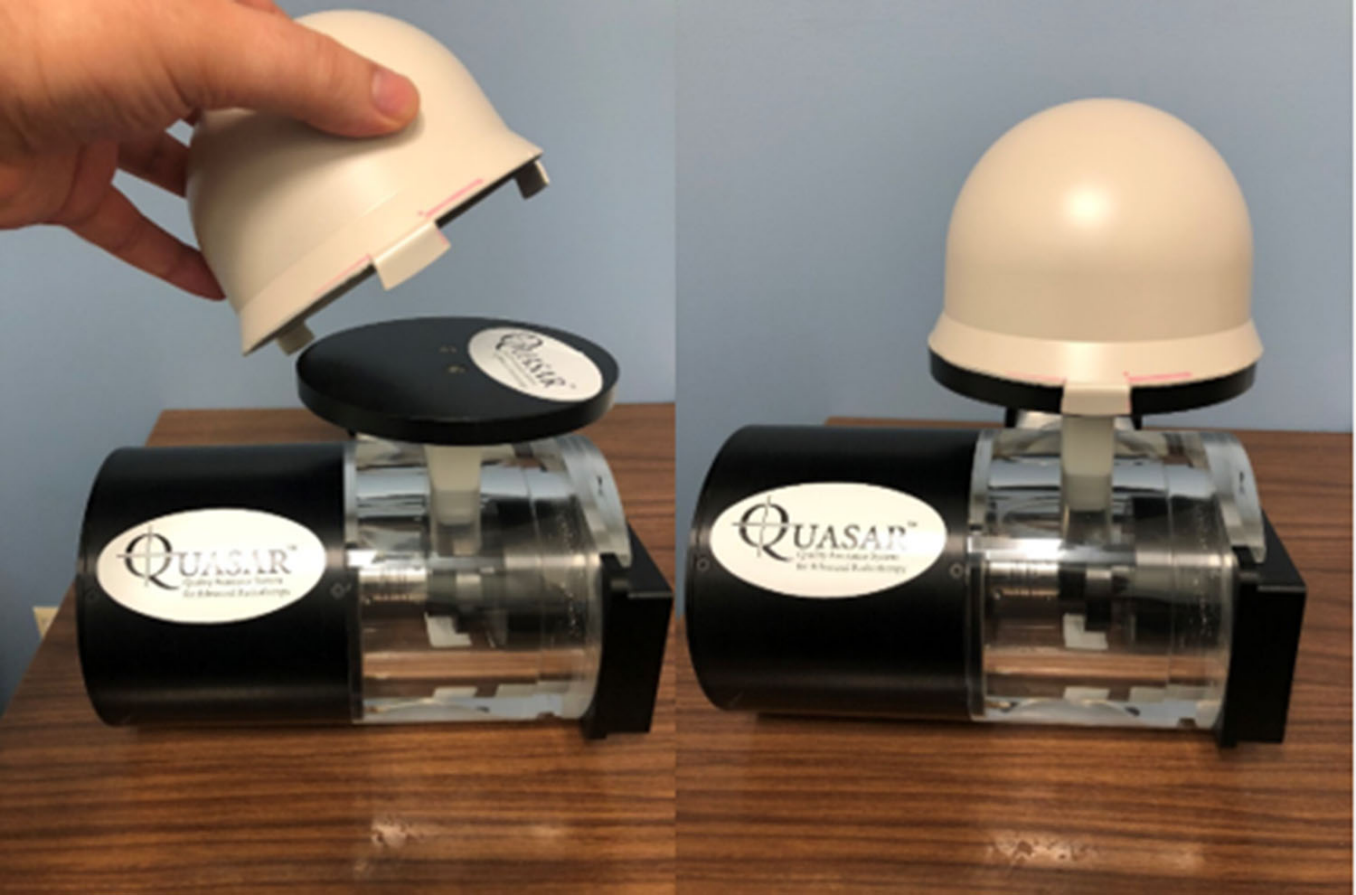
A home‐made breast phantom (in beige) can be attached to the flat chest wall phantom (in black) for enhanced surface tracking of respiratory motion.

Dynamic gating tests were completed as outlined in TG‐147. This section discusses the latency and dosimetric accuracy procedures. A specific phantom called the QUASAR Respiratory Motion Phantom (Modus Medical Devices, London, Ontario, Canada) was used for the dynamic gating tests. The QUASAR phantom includes a flat chest wall platform that is used to simulate respiratory motion in the anterior/posterior direction in conjunction with motion‐tracking systems. A home‐made breast phantom was retrofitted to the QUASAR phantom to provide larger 3D topography for surface tracking during gating tests. Figure 5 shows the QUASAR flat chest wall phantom with the in‐house breast phantom attached.

The rate of respiratory motion in the QUASAR phantom was adjusted to 18 breaths per minute (bpm). A region of interest (ROI) was drawn to cover the entire 3D surface of the in‐house breast phantom. Gafchromic films were placed in the film insert of the QUASAR phantom. By design, the motion of the flat chest wall phantom is correlated with that of the film insert. The correlation between the breast phantom and the film insert was set to 1 versus 3 cm. That is, when the film insert traveled 3 cm in a full excursion, the breast phantom traveled 1 cm in the posterior/anterior direction. When the phantom was adjusted to a 50% breathing phase at the start, the insert would travel ±1.5 cm in the superior/inferior direction as the breast phantom moved up/down by ±0.5 cm. An *X* = 3 cm by *Y* = 3 cm radiation field was delivered to the film to constitute the static non‐motion delivery. For the motion delivery, an *X* = 3 cm by *Y* = 1 cm field was delivered to the film insert. The two static and motion delivery fields were created using a uniform pattern of proton beam spots from clinically relevant region with 20‐cm range and 5‐cm modulation. Because the full excursion was 3 cm in the inferior/superior direction, it was expected to see an *X* = 3 cm by *Y* = 4 cm field on the film. A gating window was set to limit the machine to deliver only an *X* = 3 cm by *Y* = 3 cm field so that the gated delivery could be compared with the static non‐motion delivery.

Dosimetric and temporal accuracy tests were conducted as explained in TG‐147, using Gafchromic films, and the results were analyzed and compared for static and gated delivery for the same irradiated area of *X* = 3 cm by *Y* = 3 cm. For the dosimetric accuracy, a perfect ROI of ±1.5 cm in *X* and *Y* was drawn on the film analysis software (OmniPro I'mRT, IBA Dosimetry America Inc.), using both static and gated delivery films, and the averaged doses over two ROIs were compared to determine the dosimetric accuracy. For the temporal accuracy, dose profiles in the *X*‐ and *Y*‐axes corresponding to the static and gated delivery were overlaid in the same film analysis software. Full‐width half‐maximum (FWHM) differences between the static and gated delivery shots were recorded to determine the temporal accuracy.

## 2 RESULTS

In this section, we demonstrate the performance of the 5‐camera SGRT system in a proton half‐ gantry room with three image isocenters.

Table 2 shows the performance parameters in three categories with the maximum absolute setup, relative shift, and isocenter tracking accuracies in 6 DoF, as explained in Section 2.3, for the three image isocenters after radiographic image calibration using the SRS cube phantom. The overall performance accuracies were 0.3 ± 0.2 mm as mean and standard deviation (STD) in translations, including vertical, longitudinal, and lateral directions and 0.1°± 0.1° as mean and STD in rotations, including yaw, roll, and pitch for all three image isocenters. The maximum deviations in translational accuracy were 0.8 mm for absolute setup, 0.2 mm for relative shift, and 0.6 mm for isocenter tracking, and the maximum deviations in rotational accuracy of were 0.2° for absolute setup, 0.1° for relative shift, and 0.1° for isocenter tracking.

**TABLE 2:** Performance parameters of the 5‐camera surface‐guided radiation therapy (SGRT) for three image isocenters

	**(1)** Max. absolute setup error at isocenter 0/1/2			**(2)** Max. relative shift error at isocenter 0/1/2			**(3)** Max. isocenter tracking error at isocenter 0/1/2		
Six degrees of freedom	ISO‐0	ISO‐1	ISO‐2	ISO‐0	ISO‐1	ISO‐2	ISO‐0	ISO‐1	ISO‐2
Vertical (mm)	0.0	0.7	0.5	0.1	0.1	0.0	0.3	0.1	0.3
Longitudinal (mm)	0.5	0.0	0.5	0.0	0.0	0.1	0.4	0.2	0.1
Lateral (mm)	0.2	0.8	0.4	0.0	0.2	0.2	0.1	0.1	0.6
Yaw (°)	0.1	0.1	0.1	0.0	0.0	0.0	0.1	0.0	0.0
Roll (°)	0.1	0.2	0.1	0.0	0.0	0.0	0.1	0.1	0.1
Pitch (°)	0.0	0.1	0.2	0.0	0.1	0.0	0.1	0.1	0.0

**FIGURE 6:**
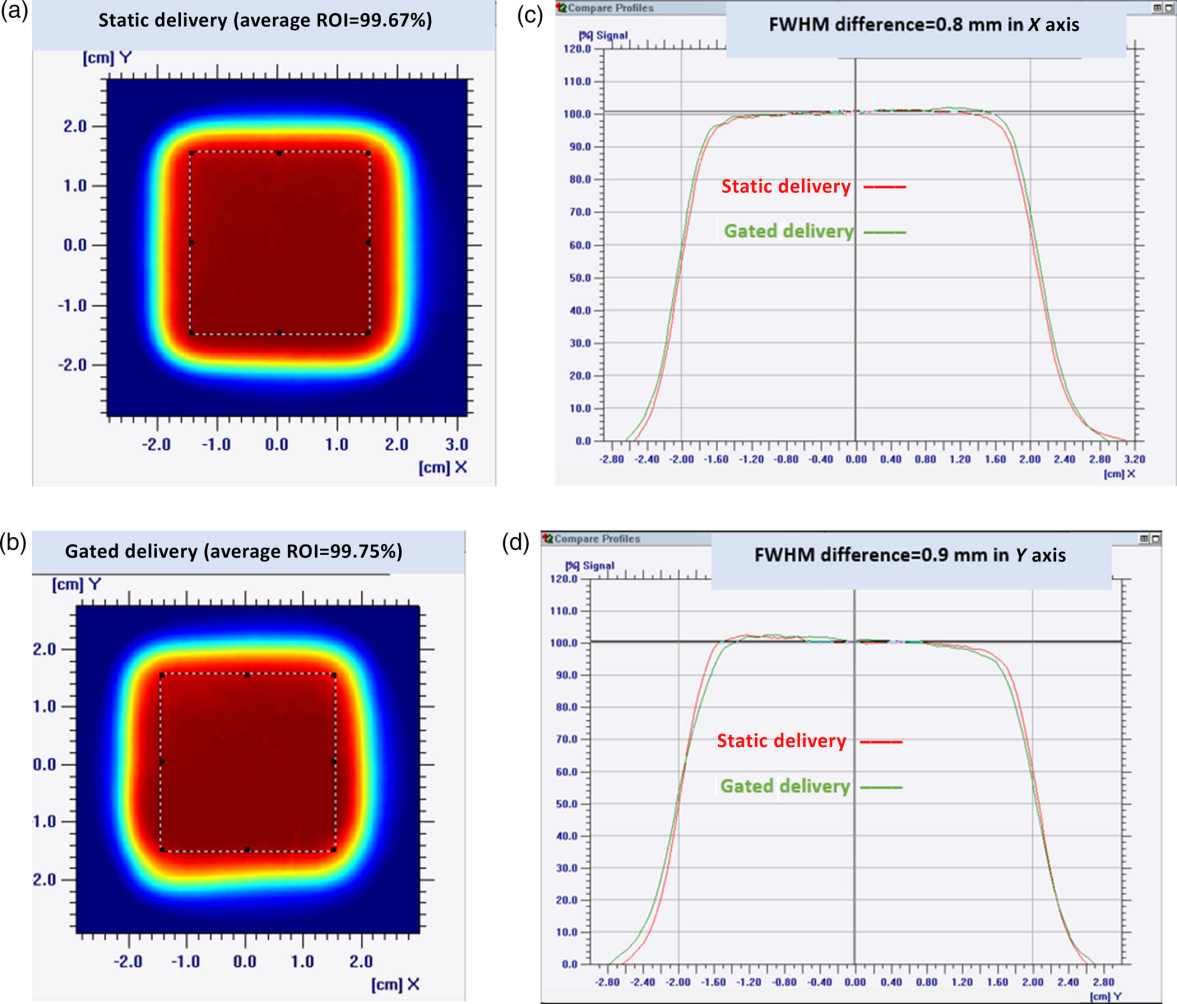
(a and b) The dosimetric and temporal accuracies of static delivery (a) and gated delivery (b) for a 3 cm by 3 cm area for dosimetric comparison. (c and D) The full‐width half‐maximum (FWHM) differences in dose profiles for the *X*‐axis (c) and *Y*‐axis (d) for temporal comparison

The dynamic gating performance of the system was measured via dosimetric and temporal accuracy tests. The tolerances of these tests were set by TG‐147 as 2% dosimetric difference and 100 ms of latency difference between tracked (gated) and non‐tracked (static) irradiation. The system resulted in a relative dose difference of only 0.1% between the gated and static fields averaged over a 3 cm by 3 cm effective area and using the ROI tool. For temporal accuracy tests, the insert was set to travel at a rate of 18 bpm. For each breath, the insert moved 3 cm in a full excursion, which corresponded to 6 cm per breath (inhalation and exhalation), totaling 108 cm/min or 1.8 cm/s. With the tolerance of 100 ms set by TG‐147, the system would have maximum positional uncertainty of 1.8 mm because of the time delay of the gating system. When the two films for the gated and static fields were analyzed, the FWHM differences were found to be 0.8 mm in *X* and 0.9 mm in *Y* with less than 50‐ms temporal accuracy. Figure 6 shows the dosimetric and temporal differences between static and gated delivery as shown on the Gafchromic films analyzed using the OmniPro I'mRT software.

## 3 DISCUSSIONS

This unique 5‐camera system was commissioned in a half‐gantry room with three distinct image isocenters. The system achieved accuracy of <1 mm in translation and <0.5° in rotation for all categories that are applicable to clinical procedures. Because there are multiple image isocenters in the half‐gantry room, the radiographic verification of the patient setup for IGRT using kV‐CBCT completed at ISO‐1 and ISO‐2 for neck‐down body cases cannot be repeated at the treatment ISO‐0 because of technical limitations. This poses a challenge for re‐verification of the patient setup after the couch is moved to the radiation isocenter at ISO‐0. The commissioned SGRT system was specifically designed to offer solutions to this problem. Once a patient is positioned at ISO‐1 or ISO‐2, their DICOM or reference surface captures can be used to align them based on the SGRT before radiographic imaging. After the kV‐CBCT is acquired and the IGRT shifts are applied, the couch will need to be moved to the treatment isocenter at ISO‐0. The advantage of the 5‐camera SGRT system is that the transition of the patient from ISO‐2 to ISO‐0 or from ISO‐1 to ISO‐0 can be monitored based on the DICOM reference or reference surface capture throughout the treatment session. A representative clinical procedure for a body case imaged at ISO‐2 and treated at ISO‐0 would be as follows:

1. Set up the patient at ISO‐2 based on the DICOM reference using SGRT.
2. Image the patient with the kV‐CBCT and apply imaging shifts to the couch.
3. Perform a reference surface capture for the patient by using SGRT while at ISO‐2.
4. Rotate the couch to the treatment ISO‐0 and verify the setup accuracy based on the SGRT reference capture acquired at ISO‐2.
5. Monitor the patient surface by using SGRT between and during beam delivery at ISO‐0.

During clinical applications, the major challenge could be ROI propagation in nonstandard SGRT systems. A reference surface capture taken in one image isocenter may not exactly propagate to another image isocenter due to nonstandard camera placements around multiple image isocenters. A remedy to this potential problem would be to slightly edit ROIs to gain optimal coverage based on the visibility of patient's surface monitored by SGRT system.

## 4 CONCLUSIONS

SGRT systems have historically been designed to accommodate a single isocenter, with the optical setup and camera systems being optimized for such a clinical environment. When additional isocenters are introduced, the existing camera system may be able to accommodate the additional locations if the distance between the isocenters is sufficiently small, such that the optical setup and camera field of view can continue to provide adequate coverage. However, when the distance between isocenters exceeds a certain value which is dependent on the room geometry and clinical applications, additional cameras and nonstandard SGRT systems may need to be considered to maintain SGRT accuracy. The proton system described in this paper is a good example of such an installation, but it is unlikely to remain unique as other treatment modalities with multiple isocenters may benefit equally from such a system. Other multiple‐isocenter systems that include setup, imaging, and treatment isocenters would benefit from “non‐standard” SGRT installations that provide SGRT coverage at all phases of setup, imaging, and treatment.

In this paper, we have outlined the need for and design of a nonstandard 5‐camera system for SGRT configurations with multiple image isocenters. The novel SGRT system featured in this report was commissioned to work with a specific type of proton device; however, the concept can be applied to any proton or photon linac that has multiple image isocenters, that has obstructed fields of view with the standard 3‐camera system, or that simply requires an improved SGRT system for better visibility and, hence, better accuracy.

## AUTHOR CONTRIBUTION

Ozgur Ates: design of the work, analysis, drafting, and final approval

Li Zhao: interpretation of data for the work, drafting, and final approval

David Sobczak: design of the work, analysis, drafting, and final approval

Fakhriddin Pirlepesov: interpretation of data for the work, drafting, and final approval

Chia‐Ho Hua: drafting the work, analysis, drafting, and final approval

Ben Waghorn: interpretation of data for the work, drafting, and final approval

Thomas E. Merchant: drafting, interpretation of data for the work, and final approval

## CONFLICT OF INTEREST

Ben Waghorn is an employee of Vision RT Inc., London, UK. There is no other conflict of interest present for the authors.

## Data Availability

The data that support the findings of this study are available on request from the corresponding author. The data are not publicly available due to privacy or ethical restrictions.
